# ^177^Lu-PSMA-617 radioligand therapy for a patient with lymph node metastatic prostate cancer

**DOI:** 10.18632/oncotarget.19805

**Published:** 2017-08-02

**Authors:** Finn E. von Eyben, Timo Kiljunen, Timo Joensuu, Kalevi Kairemo, Christian Uprimny, Irene Virgolini

**Affiliations:** ^1^ Center of Tobacco Control Research, Odense, Denmark; ^2^ Docrates Cancer Center, Helsinki, Finland; ^3^ Department of Nuclear Medicine, Innsbruck University Hospital, Innsbruck, Austria

**Keywords:** abiraterone, cross-over study, external beam radiotherapy, lutetium radiolabled prostate membrane specific antigen radioligand therapy, metastatic prostate cancer

## Abstract

Prostate specific membrane antigen (PSMA) is expressed in unfavorable prostate cancer. PSMA is basis for new diagnostics and theranostics. PET/CT using PSMA is more sensitive than choline PET/CT. ^177^Lu-PSMA radioligand therapy is mainly used for patients with end-stage prostate cancer. This report describes a patient with a third recurrence in lymph nodes. The recurrence was treated with ^177^Lu-PSMA radioligand therapy instead of chemotherapy with docetaxel. The effect was in part evaluated relative to that of two established salvage treatments. Prior salvage radiotherapy and abiraterone of the first and second recurrence in lymph nodes had given only a partial reduction of PSA. Nevertheless within five months of follow-up, ^177^Lu-PSMA radioligand therapy of the third recurrence in lymph nodes reduced PSA for a period to unmeasurable levels. ^177^Lu-PSMA radioligand therapy gave only mild adverse effects. In conclusion, for a patient with lymph node metastatic prostate cancer, ^177^Lu-PSMA-617 radioligand therapy had an attractive therapeutic profile. A follow-up study of similar patients is being planned.

## INTRODUCTION

Up to a third of patients with prostate cancer (PC) who undergo radical prostatectomy (RP) later develop a continuous rise of prostate specific antigen (PSA). A low but rising PSA level has been denoted biochemical recurrence (BCR). In this phase of recurrence, conventional imaging do not detect the sites of recurrence. Patients with BCR may be treated with salvage external beam radiotherapy (SRT) with or without androgen deprivation therapy (ADT) [1, 2]. Five treatments prolong survival for patients with metastatic castration-resistant (mCR) PC.

Most poor-risk PC express prostate membrane specific antigen (PSMA). Correspondingly, ^68^Ga-PSMA HBED-CC PET/CT detected cancer lesions for most of these patients [3]. ^177^Lu-PSMA-617 radioligand therapy (RLT) was effective for patients with PSMA-positive mCRPC [4]. Guidelines described the best practice for ^68^Ga-PSMA HBED-CC PET/CT and ^177^Lu-PSMA-617 RLT [5, 6]. However, ^177^Lu-PSMA-617 RLT is mainly used for patients with end-stage mCRPC who had failed with established systemic treatments.

We report a patient who had three episodes of recurrence with lymph node metastatic prostate cancer. The first two episodes had been treated with SRT and abiraterone. The third episode was treated with ^177^Lu-PSMA-617 RLT.

## RESULTS

**Figure 1:**
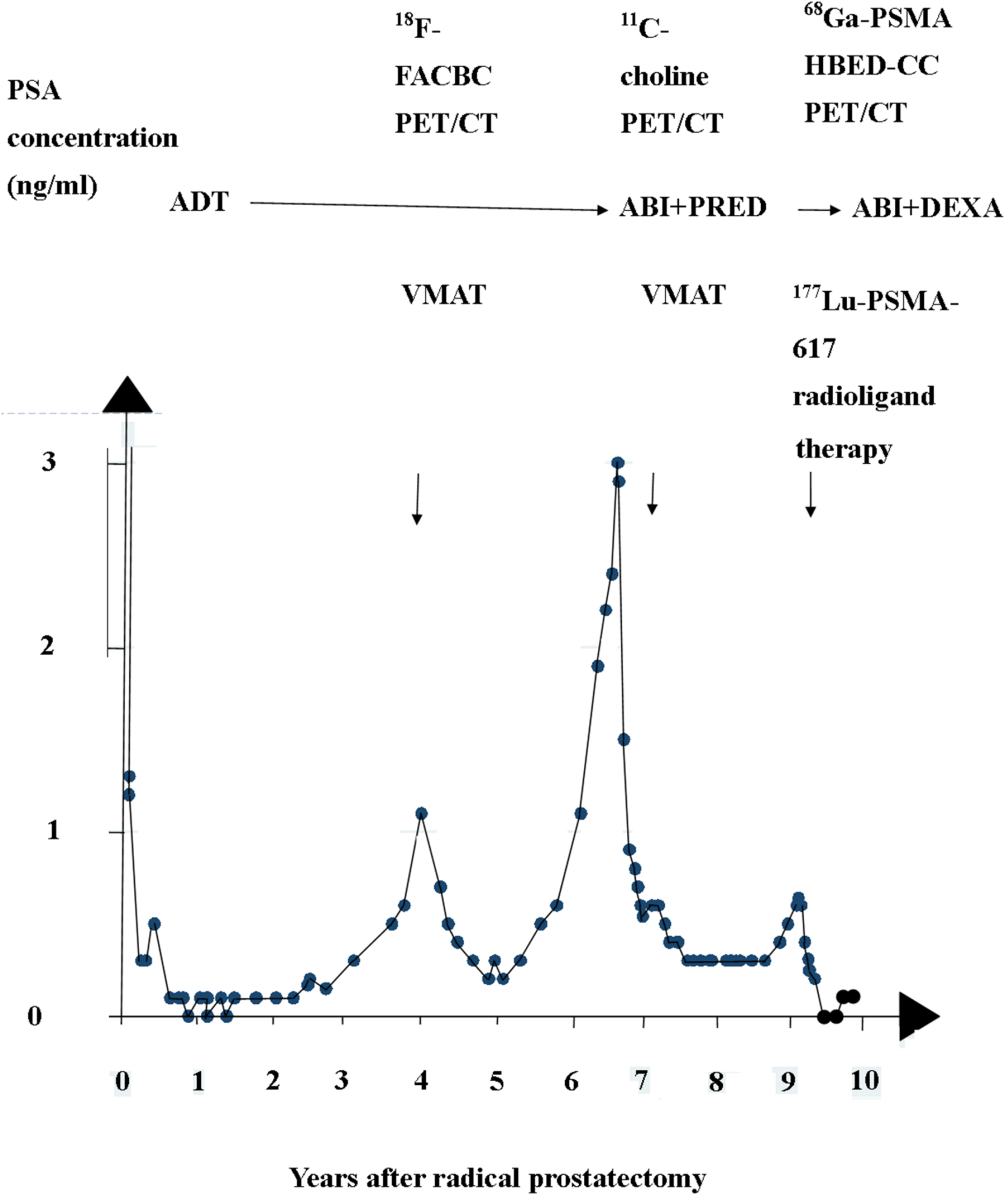
Three episodes of rising PSA after radical prostatectomy The two first episodes were treated with volume modulated arc therapy (VMAT), and abiraterone and prednisone (ABI + PRED). The combination was later shifted to abiraterone and dexamethasone (ABI+DEXA). The third episode was treated with ^177^Lu-PSMA-617 RLT.

A 50-year-old patient was diagnosed with PC in May 2007. At diagnosis, he underwent RP and limited bilateral pelvic lymphadenectomy (pT3b, pN1 M0; Gleason score 9 (4+5)). He started androgen deprivation therapy (ADT) shortly after RP. Two years later, he had continuously rising PSA levels (Figure 1). With progression of PSA, PSA doubling time became reduced to four months. In 2011, an anti-1-amino-3-^18^F-fluorobutane-1-carboxylic acid (^18^F-FACBC) PET/CT showed one lesion in a pelvic lymph node. He was given SRT with a boost for the lesion. The boost was given with 2 Gy per fraction and a conventional fractionation scheme to a cumulative dose of 78 Gy. A follow-up PET/CT showed that the patient had achieved a partial remission [7]. Nadir PSA after SRT was 0.2 ng/ml. In 2013, he again had rising PSA. In 2014, a new restaging with ^11^C-choline PET/CT was carried out. The new PET/CT showed he had developed a new site in a retroperitoneal lymph node. Therefore his treatment was changed to abiraterone 150 mg a day and prednisone 5 mg twice daily. Three months later, the patient also underwent a second course of SRT with a cumulative dose of 60 Gy. The second SRT was targeting the new site. Again, the combined treatment transitorily reduced PSA. The new nadir PSA was 0.3 ng/ml.

**Figure 2:**
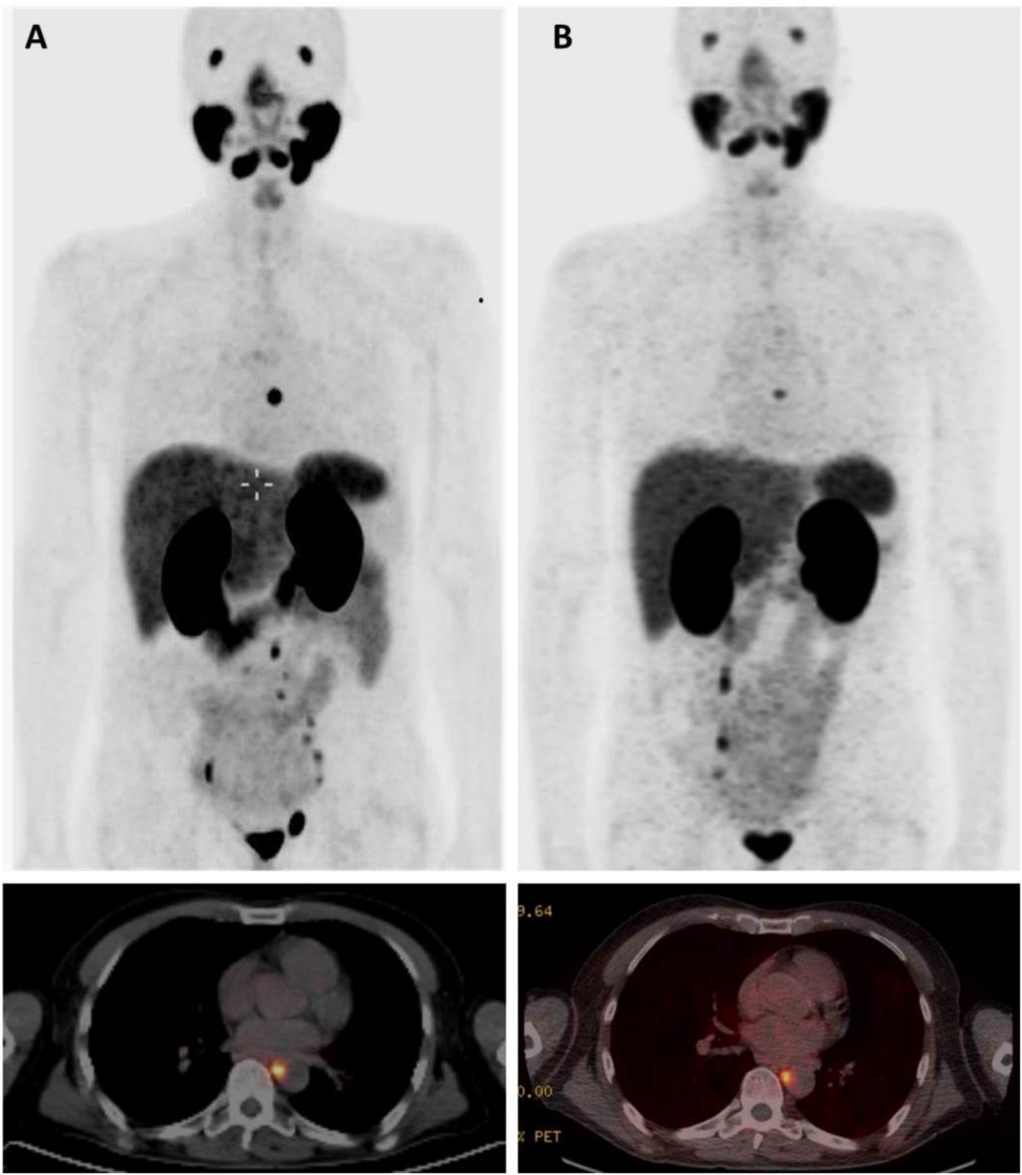
^68^Ga-PSMA-HBED-CC PET/CT before and after treatment with ^177^Lu-PSMA-617 RLT **Panel A** shows PET/CT before treatment July 2016 with the strongest lymph node uptake in a paraesophageal lymph node and uptakes in lymph nodes in the obturatory regions and sites in left internal and common iliac nodes. **Panel B** shows PET/CT after treatment November 2016 with uptake only in the mediastinal lymph node.

In 2016, the patient had a third episode of continuously rising PSA. A restaging ^68^Ga-PSMA HBED-CC PET/CT in July 2016 showed new pathologic uptakes in a mediastinal lymph node and several infradiaphragmal lymph nodes (Figure 2A). At that time, he had an ECOG performance status of 0, he had no pain, and had normal levels of thrombocytes. PSA was 0.6 ng/ml. However, the patient preferred to be treated with ^177^Lu-PSMA-617 RLT. He wanted to postpone chemotherapy with docetaxel. In July and September 2016, he was given two cycles of ^177^Lu-PSMA-617 RLT with a radioactivity of 6 gigabequerel (GBq) in both cycles [6]. The interval between the two cycles was eight weeks. Dosimetric analyses indicated a cumulative tumor dose of 37.7 Gy. The dose corresponded to a dose (EQD2) of 226 Gy. We calculated the EQD2 for external beam radiotherapy given with a standard fractionation of 2 Gy per fraction and with alfa/beta ratio for PC estimated as 1.5. The first cycle of ^177^Lu-PSMA-617 RLT caused acute nausea. But with use of anti-emetic drugs, the patient avoided nausea in connection with the second cycle. Otherwise the ^177^Lu-PSMA-617 RLT did not cause acute adverse effects.

Follow-up ^68^Ga-PSMA HBED-CC PET/CT was carried out in November 2016 and February 2017 (Figure 2B). In the follow-up, the mediastinal lymph node showed a reduction of SUVmax from 21.6 to 8.6. All other pathologic sites had normalized. Total tumor volume had decreased from 18 cm^3^ to 1.5 cm^3^. From December 2016 to May 2017 PSA has been unmeasurable. In December 2016, the combination of abiraterone-and prednisone was switched to a combination of abiraterone 150 mg per day and dexamethasone 1 mg per day. In May and June 2017, he had a slight rise of PSA. Until June 2017, the patient had no chronic adverse effects.

## DISCUSSION

We report treatment with ^177^Lu PSMA-617 RLT for a patient with a third episode of lymph node metastatic prostate cancer. The treatment had a positive balance between effects and adverse effects. The ^177^Lu-PSMA-617 RLT reduced pre-treatment PSA more than SRT and abiraterone. Also studies of patients with end-stage PC treated with ^177^Lu PSMA-617 RLT had reported a positive balance between effects an adverse effects [4].

Restaging with conventional imaging for the patient did not reveal sites of recurrence at the three episodes of continuously rising PSA Hence, restaging with conventional imaging would have concluded that the patient had biochemical recurrence. However, restaging with PET/CT with relevant radiotracers showed sites in abdominal and thoracic lymph nodes. The SRT was guided by the PET/CT findings. The clinical course after the guided SRT rules out that the PET/CT showed false-positive findings. Therefore the correct restaging for the patient at the time of the rising PSA levels was lymph node metastatic PC. Present guidelines recommend that clinicians include choline PET/CT in restaging of patients with rising PSA after RP.

According to guidelines, SRT should be started while PSA is <0.5ng/ml [2]. Previously choline PET/CT rarely gave positive findings while rising PSA levels were so low. That may have contributed to the patient had PSA levels > 1 ng/ml at the time of restaging PET/CTs. However today, ^68^Ga-PSMA HBED-CC PET/CT detects sites of recurrence for half of the patients who have rising PSA <0.5 ng/ml.

The ^177^Lu-PSMA RLT had given 48 mSv for each cycle according to dosimetric analyses in a previous study [8]. The radiation implies a class III risk of cancer according to the classification of the International Commission of Radiation Protection. We estimate the life-time risk for a 50-year old individual for developing cancer to be 25% without lutetium RLT. The estimated risk increased to 25.5% with the lutetium RLT.

Radiotherapy has a dose-response relation for PC [1]. The two previous series of SRT for the patient had been given with a total boost dose of 78 Gy and 60 Gy whereas ^177^Lu-PSMA RLT was given with a calculated E2Q dose of 226 Gy. Therefore ^177^Lu-PSMA-617 RLT reduced PSA more than the two previous courses of SRT. The previous SRT had limited effect despite the restaging had used the best available PET/CT and the SRT was guided by PET/CT. For the patient, the second and third recurrence of lymph node metastatic prostate cancer had started as micrometastases proximal to the radiation field of the two courses of SRT. The micrometastases were not detected by the PET/CTs. Anyway, an increasing number of studies report that SRT has been guided by ^68^Ga-PSMA-HBED-CC PET/CT [9–12].

In the third episode of rising PSA, the patient had four measurements with a progressive rise of PSA while he was treated only with abiraterone. Therefore abiraterone did not contribute to the fall of PSA after ^177^Lu-PSMA-617 RLT was started. The patient had the massive reduction of PSA to unmeasurable values in a period from December 2016 to May 2017. In December 2016, the combination of abiraterone and prednisone was switched to a combination of abiraterone and dexamethasone, Therefore dexamethasone did not contribute to the massive reduction of PSA after he was given ^177^Lu-PSMA-617 RLT.

A new study of patients with lymph node metastatic castration-resistant PC treated with ^177^Lu-PSMA-617 RLT is being planned. The study is submitted for the regional science-ethics committee of Southern Denmark. Also a systematic review of mCRPC is being planned that compares the therapeutic profile of third-line treatment and ^177^Lu-PSMA-617 RLT (Prospero database, CRD 42017067743).

In conclusion, our study is a good example of clinical efficacy with ^177^Lu-PSMA RLT for lymph node metastatic castration-resistant PC.
